# Urease Inhibitory Kinetic Studies of Various Extracts and Pure Compounds from Cinnamomum Genus

**DOI:** 10.3390/molecules26133803

**Published:** 2021-06-22

**Authors:** Manoj Kumar, Neha Sikri, Sulekha Chahal, Jitender Sharma, Bhavna Sharma, Poonam Yadav, Monika Bhardwaj, Divya Vashishth, Pooja Kadyan, Sudhir Kumar Kataria, Sunita Dalal

**Affiliations:** 1 Department of Biotechnology, Kurukshetra University, Kurukshetra 136119, India; manojkumar@kuk.ac.in (M.K.); Sikrineha3@gmail.com (N.S.); schahalbiotech@kuk.ac.in (S.C.); jksharmakuk@rediffmail.com (J.S.); bsharma2744@gmail.com (B.S.); 2 Department of Zoology, Maharshi Dayanand University, Rohtak 124001, India; poonambalewa93@gmail.com (P.Y.); bhardwajmoni92@gmail.com (M.B.); vashdiv20@gmail.com (D.V.); pooja.kadyan546@gmail.com (P.K.); sudhir.zoology24@mdurohtak.ac.in (S.K.K.)

**Keywords:** urease inhibition, Cinnamomum, camphene, cuminaldehyde

## Abstract

Urease is an enzyme that plays a significant role in the hydrolysis of urea into carbonic acid and ammonia via the carbamic acid formation. The resultant increase in pH leads to the onset of various pathologies such as gastric cancer, urolithiasis, hepatic coma, hepatic encephalopathy, duodenal ulcers and peptic ulcers. Urease inhibitors can reduce the urea hydrolysis rate and development of various diseases. The Cinnamomum genus is used in a large number of traditional medicines. It is well established that stem bark of *Cinnamomum cassia* exhibits antiulcerogenic potential. The present study evaluated the inhibitory effect of seven extracts of *Cinnamomum camphora*, *Cinnamomum verum* and two pure compounds Camphene and Cuminaldehyde on urease enzyme. Kinetic studies of potential inhibitors were carried out. Methanol extract (IC_50_ 980 µg/mL) of *C. camphora* and a monoterpene Camphene (IC_50_ 0.147 µg/mL) possess significant inhibitory activity. The Lineweaver Burk plot analysis suggested the competitive inhibition by methanol extract, hexane fraction and Camphene. The Gas Chromatography-Mass Spectroscopy (GC–MS) analysis of hexane fraction revealed the contribution of various terpenes. The present study targets terpenes as a new class of inhibitors that have potential therapeutic value for further development as novel drugs.

## 1. Introduction

Enzyme inhibition studies are a significant area of research in pharmaceutical studies, as they result in the discovery of drugs for the treatment of various physiological conditions. Urease is a highly efficient catalyst for the hydrolysis of urea with a rate approximately 10^14^ times the rate of non-catalyzed reaction. Urease has been exhaustively studied and is considered the basis of biochemistry as it was the first enzyme to be crystallized. The three-dimensional structure of this Ni^2+^-dependent hydrolytic enzyme, from various sources has been elucidated [1]. The similarity in amino acid sequence from various origins suggests a common ancestry of urease [2].

Urease activity is widespread among the prokaryotes. The enhanced activity of urease leads to various conditions such as pathological changes in humans infected with *Helicobacter pylori*, *Proteus mirabilis*, *Ureoplasma urealyticum, Klebsiella pneumonia*, *Staphylococcus* and *Salmonella* species. *H. pylori* prevails in 50% of the global population, with or without disease symptoms. The urease enzyme in cytoplasm and/or on the surface of *H. pylori* is a major virulence factor and is implicated for its high prevalence in the human population [3]. *H.* *pylori* infection leads to gastric inflammation, duodenal and gastric ulcers, gastric adenocarcinoma and gastric lymphoma. The ureolytic activity of *P. mirabilis*, *U. urealyticum*, *K. pneumoniae*, *Staphylococcus* and *Salmonella* species is a major virulence factor responsible in the pathogenesis of many other clinical conditions such as hepatic coma, pyelonephritis and urinary stones [4,5].

**Scheme 1:**
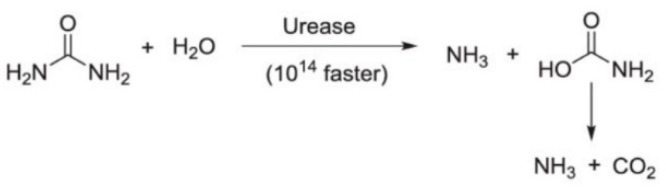
The carbamate decomposes spontaneously into a second molecule of ammonia and bicarbonate.

The basic mechanism in all these conditions is urea hydrolysis by ureolytic microorganisms in the stomach, gastrointestinal tract (GI) as well as in the urinary tract. Here, the rapid hydrolytic decomposition of urea occurs, producing ammonia and carbamate in the presence of urease. The carbamate eventually decomposes spontaneously into a second molecule of ammonia and bicarbonate (Scheme 1). The increase in pH favors various human pathogenic bacteria which exploit urease as a virulence factor to further infect and colonize the host. Therefore, the slowing down of urea hydrolysis may reduce the ailments, ammonia loss and improves urea utilization in agricultural soils as well. Urea has been universally accepted as an inexpensive protein source for various ruminants and non-ruminants. Enhanced urease activity by GI microorganisms in ruminants fed with urea causes damage to GI mucosa, resulting in impaired nutrient absorption, protein spillage and decreased growth performance [6].

Plants of the Lauraceae family have been widely reported as gastro protective [7,8], antiulcerogenic [9], stomach disorder protectant, in treating kidney infection and kidney stones [10]. Traditionally, the plants of this family are used to treat gastrointestinal spasms for digestive disorders such as indigestion, bloating gas, etc.

Crude extracts and constituents from about thirty species of Cinnamomum displayed significant activities, namely antibacterial, anticancer, antifungal, antiseptic, antiviral, anti-inflammatory, antipyretic, antioxidant, chemopreservative, cytotoxic, anti-diabetic, antiulcer, immune stimulant, etc. Compounds such as Camphene, Cuminaldehyde, Eugenol, Proanthocyanidins etc., have been reported to be responsible for these activities [11,12,13]. The compounds Camphene and Cuminaldehyde exhibit antibacterial and antifungal properties [14]. Treatment with Camphene exhibited no toxicity in human hepatic cells; also, it has been reported to have antitumor activities [15]. Camphene exhibits antiulcer activity and was found to be helpful in the expulsion of urolithiasis [16]. A binational, double-blind, placebo-controlled randomized clinical trials study reports improvement in the blood sugar of people with prediabetes and delayed progression to type 2 diabetes when treated with the cinnamon [17]. The essential oils, extracts, and the main components Cinnamaldehyde, Eugenol and Linalool from cinnamon show significant antimicrobial activities against oral pathogens and could be beneficial in caries and periodontal disease prevention, endodontics, and candidiasis treatment [18]. Another biologically significant compound, 2-methoxycinnamaldehyde from *C. verum*, proved a novel antiproliferative drug, inducing cell death through targeting both topoisomerase I and II in human colorectal adenocarcinoma COLO 205 cells [19].

*C. verum, C. cassia, C. zeylanicum, C. camphora* and *C. cosphoeum* are the only five species that have been investigated scientifically for various biological activities from 300 available Cinnamomum species [20]. The majority of the studies have been focused on the essential oils and aqueous/alcohol extracts in leaves and stem barks, with the main focus on validating the uses of Cinnamomum in various traditional systems. There are reports of *C. verum* bark used as spice for various ailments and particularly related to stomach disorders and in ulcer treatments [21].

Novel and efficacious urease inhibitors with good bioavailability and low toxicity are significant, especially in third world countries with high infection rates of *H. pylori* [22]. In the present study, various samples obtained from Cinnamomum species such as leaves (*C.* *camphora*), bark (*C. verum*) and two pure compounds found in significant proportion in the Cinnamomum species, i.e., Cuminaldehyde (an aldehyde) and Camphene (a monoterpene) were studied for urease inhibition potential. The work is focused on seeking novel natural urease inhibitors from herbal sources that can be used directly or as a lead compounds in the management of urease-related complications.

## 2. Results and Discussion

### 2.1. Effect of Plant Extracts and Compounds on Urease Inhibition

The methanol and aqueous extracts of the *C. camphora, C. verum* and the compounds Camphene and Cuminaldehyde showed varied urease inhibitory potential. Methanol extract of *C. camphora* and Camphene obtained inhibitory potential closer to the standard, Thiourea. Sub fractions of *C. camphora* in three different solvents, i.e., hexane, chloroform and ethyl acetate, were prepared as described in the Materials and Methods section. The sub fractions of *C. camphora* were also studied for their urease inhibitory potential. Among them, hexane and ethyl acetate sub fractions were further studied to determine the mode of inhibition because they had a significant inhibitory effect on urease (Table 1).

### 2.2. Urease Inhibition Curve Analysis

**Figure 1:**
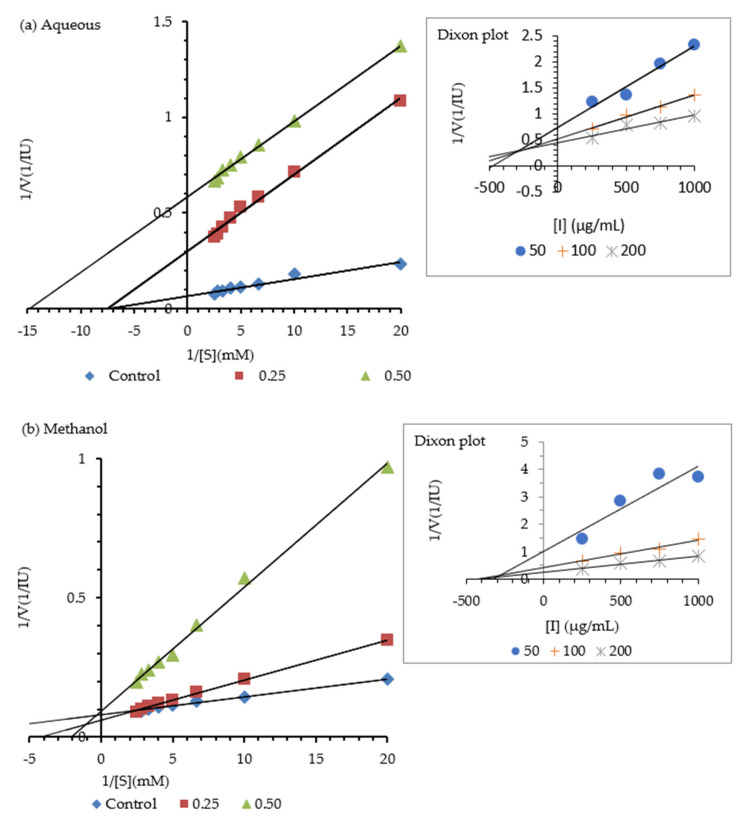

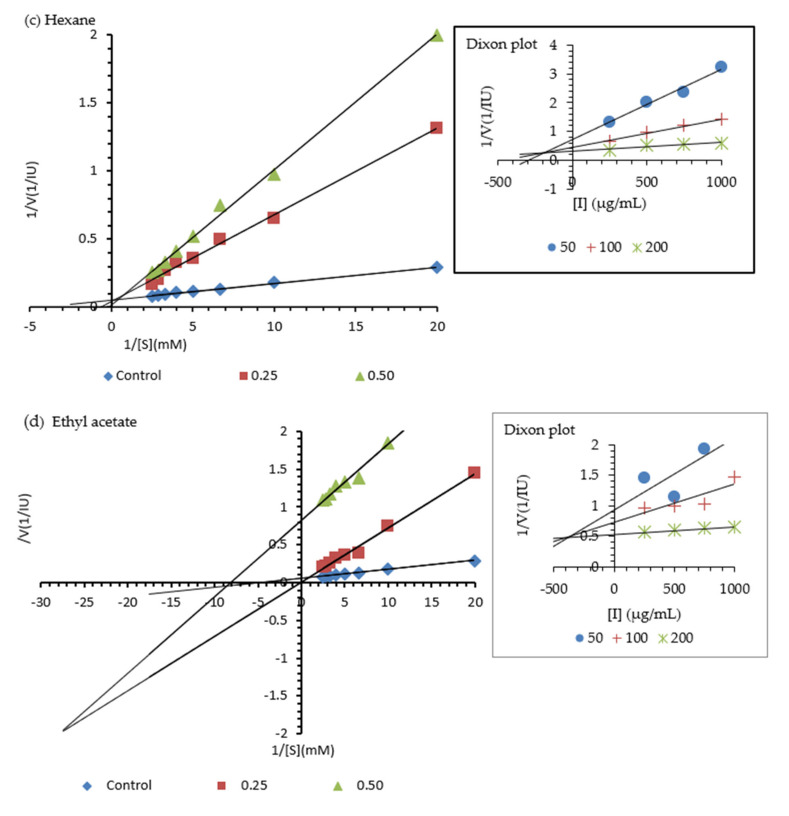

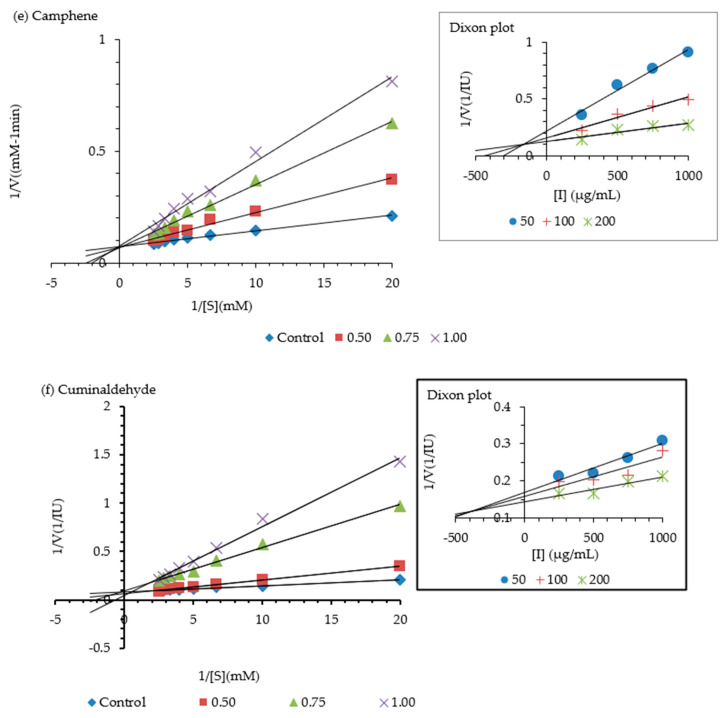
Representative Lineweaver Burk Plots for inhibition of urease enzymatic activity by different concentrations of extracts of *C. camphora* (mg/mL) (**a**–**d**), Camphene and Cuminaldehyde (mM) (**e**,**f**). Representative Dixon Plots as insert graphs of respective samples at different concentrations of urea (µM).

The assessment of urease inhibition by the various samples (extracts/compounds) is shown in Table 2. The inhibitory activity was found to increase linearly with an increase in the concentration of studied samples. The trend of lines for different concentrations of investigated samples gave an idea about the mode and mechanism of inhibition. In the case of Hexane fraction (Figure 1c) and Camphene (Figure 1e), the set of lines were found to intersect each other at the same point on the Y-axis, which depicted that V_max_ was unchanged and there was a decrease in Km. Hence, they exhibited the reversible, competitive inhibition of the urease enzyme. Meanwhile, in the aqueous extract (Figure 1a), ethyl acetate fraction (Figure 1d) and Cuminaldehyde (Figure 1f) a decrease in V_max_ and lines meeting at a point away from the Y-axis was seen. They are considered as a mixed type of inhibition. The Dixon plots determined inhibitory constants (K_i_), at the intersection across the X-axis, with minimum values of Hexane fraction and Camphene (Table 2).

### 2.3. GC–MS Analysis

The chemical composition of the potential urease inhibitor, i.e., hexane fraction, was analyzed by GC–MS. It resulted in the identification of thirty-eight compounds. Out of the total constituents, more than 30% of total contributions to terpenes are mainly gamma sitosterol: a triterpenoid (17.23%), phytol:acyclic diterpene (8.37%), 3,7,11,15-Tetramethyl-2-hexadecen-1-ol:acyclic diterpene (1.49%), solanesol: terpene, (1.17%), squalene: acyclic triterpene (0.65%), camphor: terpenoid. Other constituents are esters, free fatty acids and vitamin B8, etc. The details of constituent compounds are provided as supplementary material (Table S1 and Figure S1).

The urease inhibitory potential of the hexane sub fraction constituting approximately 30% of various terpenes exhibited a competitive mode of inhibition with low K_i_. The results imply a synergism among these terpenes. The valid role of terpenes in urease inhibition is further confirmed by Camphene and Cuminaldehyde. Camphene, a bicyclic monoterpene, revealed a competitive mode of inhibition, as close to standard thiourea. Cuminaldehyde, an oxidized aldehyde monoterpene, also appeared as a potential urease inhibitor, although it exhibited mixed inhibition. The hexane fraction has compounds which displayed the fact that competitive inhibition needed to be further evaluated for the development of the effective drug against urease enzyme.

The conventional method for the treatment of ureolytic bacterial infections is by the use of antibiotics, but the drawback is the development of antibiotics resistance. The suggested combined treatment with urease inhibitors and antibiotics would allow the use of low doses of antibiotics and the prevalence of antibiotics resistance in patients with kidney, ulcer treatments, etc. Many reports have revealed the role of terpenes as urease inhibitors from various plants. Myrsinol (diterpene) has been studied and reported as an uncompetitive urease inhibitor from the plant *Euphorbia decipiens* [23] with K_i_ = 117.40 µM. Another terpene, Vernonione, from the roots of plant *Vernonia cinerascens*, exhibited IC_50_ = 227.6 µM [24], and was accounted as a good urease inhibitor. Atranorin is a monoterpene from stem bark of *Stereospermum acuminatissimum K. Schum* with IC_50_ = 18.2 ± 0.03 μM with excellent urease inhibition potential [25]. The Cinnamomum genus is well documented for stomach disorders. This is the first report on analyzing the urease inhibitory potential of the Cinnamomum genus, Camphene and Cuminaldehyde. Camphene competitively inhibited the urease enzyme with a K_i_ value of 1.431 mM. In future studies, the various compounds of the different extracts will be singularly evaluated, which will allow for K_i_ determination specifically.

## 3. Material and Methods

### 3.1. Collection of Plant Material

The two plant species (*Cinnamomum camphora* (L) *J. Presl* and *Cinnamomum verum*) of a family Lauraceae were selected based on their pharmacological reports and traditional uses. The leaves of the *C. camphora* were collected in the months of August–September, 2017 from the campus of Kurukshetra University, Kurukshetra, India and were identified by Prof. B.D. Vashistha, Department of Botany, Kurukshetra University. The bark of the *C. verum* was purchased from the market. A voucher specimen (Herbarium/Bot.Ku/Biotech-2-2017) of *C. camphora* and *C. verum* (Herbarium/Bot.Ku/Biotech-3-2017) were deposited in a herbarium. The plant materials were washed and rinsed with distilled water repeatedly to remove any soil or solid particulates and shade dried at 40 °C for 2 days. After drying, the sample was ground to a fine powder using a mechanical blender and stored for further use, before extraction.

### 3.2. Chemicals

Urease (Type IX from *Canavalia ensiformis* (Jack Bean), specific activity: 50,000–100,000 units/g), compounds Camphene (CID: 6616) and Cuminaldehyde (CID:326) were purchased from Sigma Aldrich (Saint Louis, MO, USA). One unit of urease enzyme is equivalent to 1.0 I.U. Other chemicals such as urea, thiourea, sodium nitroprusside and phenol were obtained from Hi-Media. All reagents were of analytical grade.

### 3.3. Extraction of Plant Material

The powder of dried leaves of *Cinnamomum camphora* and bark of *Cinnamomum verum*, each 10 g, was separately soaked in sterilized distilled water and 99% methanol (100 mL each) in a reagent bottle covered with a lid at 37 °C for 24 h. Each soaked powder was packed into a soxhlet column for 48 h. The water and methanol extracts were evaporated and concentrated to dryness using the rotary evaporator at 50 °C. Based on the results of % Inhibition and IC_50_ values of the extracts; the methanol crude extract was dissolved in methanol–water (5:5, *v/v*), followed by liquid–liquid fractionation using *n*-hexane, chloroform and ethyl acetate, respectively. The fractions were dried and stored at −4 °C for further use.

### 3.4. Measurement of Urease Inhibitory Activity

For the initial urease inhibitory activity, a 1 mg/mL concentration of all investigated samples were prepared. The assay solution mixture comprising 1.2 mL of 10 mM phosphate buffer solution containing 10 mM lithium chloride and 1 mM ethylene-diamine-tetra acetic acid, pH 7.2 at 37 °C, 0.2 mL (1 mg/5 mL) of urease enzyme solution and 0.1 mL of each sample was subjected to 5 min incubation. A total of 0.5 mL (400 μM) of substrate was added. After 20 min of incubation at 27 °C, 1 mL of solution A (contained 0.5 g of phenol and 2.5 mg of sodium nitroprusside in 50 mL of distilled water) and 1 mL of solution B (contained of 250 mg sodium hydroxide and 820 μL of sodium hypochlorite 5% in 50 mL of distilled water) was added. Urease activity was determined by measuring the ammonia released during the reaction at 640 nm, using the modified method described by Weatherburn [26].

Thiourea was assayed as the standard inhibitor compound.

I% = 100 − (T/C × 100)(1)

where I% is the percent inhibition of the enzyme, T (test) is the absorbance of the tested sample in the presence of enzyme, C (control) is the absorbance of the solvent in the presence of enzyme. The activity of uninhibited urease was chosen as the control activity of 100%.

### 3.5. Statistical Analysis

All the assays were carried out in triplicates to test the reproducibility. The results are presented as mean ±S.E.M. SPSS 15.0 was used for statistical analysis. The values of *p* < 0.05 were considered statistically significant. Correlations among data obtained were calculated using Pearson’s coefficient (r).

### 3.6. Determination of Kinetics Parameters

The concentration that induces an inhibition halfway between the minimum and maximum response to each sample (IC_50_) was determined by analyzing the inhibitory effect of samples at various concentrations in the assay. After monitoring urease activities in the absence and presence of two different concentrations of extracts (0.25 mg/mL and 0.50 mg/mL), and three different concentrations (0.50 mM, 0.75 mM and 1.00 mM) of Camphene and Cuminaldehyde, Lineweaver Burk graphs were prepared for the determination of inhibition type. Inhibitory constants (K_i_), from the Dixon plots were determined as the intersection on or corresponding to the X-axis of the plots of 1/V vs. [I]. The plot represents different concentrations of potential inhibitory samples at 50 µM, 100 µM and 200 µM urea concentrations. All experiments were conducted in triplicate.

### 3.7. GC–MS Analysis

The GC–MS technique was used for identifying bioactive compounds in the *C. camphora* hexane fraction having the significant urease inhibitory potential. The sample was analyzed using a Shimadzu Mass Spectrometer-QP 2010 Plus. The instrument was equipped with a split injector and a Perkin Elmer Auto system XL gas chromatograph interfaced with a turbo-mass spectrometric mass selective detector system. A total of 1 μL of the sample was injected in GC–MS and mass spectroscopy (MS) was operated in EI mode (70 eV) with helium as carrier gas at a flow rate of 1.21 mL/min. The analytical column Rtx-5 capillary column (length—60 m × 0.25 mm i.d., 0.25 μm film thickness) was used for the separation of compounds. The column temperature was programmed from 60 °C to 300 °C at withhold times of 2 and 28 min, respectively, and column head pressure was adjusted to 73.3 kPa. A solvent cut time of 8 min was adjusted. The injector temperature was set at 270 °C and GC–MS interface was maintained at 280 °C. The MS was operated in the Acquisition mode (ACQ) mode scanning from *m*/*z* 40 to 700. In the full scan mode, electron ionization (EI) mass spectra 134 in the range of 40–700 (*m*/*z*) was recorded at electron energy of 70 eV. By comparing mass spectra with the library of the National Institute of Standard and Technology (NIST), USA/Wiley different compounds were identified.

## 4. Conclusions

The findings of urease inhibition studies based on Lineweaver Burk and Dixon plot analysis of *C. camphora* (hexane fraction) and Camphene supports them as potent inhibitors with competitive mechanisms. The inhibition analysis presented here can be beneficial in dose quantification. The findings of the present investigation also provide a scientific support towards the traditional uses of plant materials under study in stomach-related diseases. The potential of investigated urease inhibitors could be the novel therapeutics and need to be explored further. It is also further concluded that Cinnamomum spps can be explored as a viable source of some natural urease inhibitory constituents.

## Figures and Tables

**Table 1:** Inhibition studies of the plant extracts/sub fractions/pure compounds against urease enzyme.

Samples *	% Urease Inhibition **	IC_50_ (µg/mL)
*C. camphora* (Aq)	35.00 ± 0.01	1340
*C. camphora* (Met)	70.07 ± 0.03	980
*C. camphora* (H.F.)	43.52 ± 0.57	1210
*C. camphora* (C.F.)	17.04 ± 1.38	N.A.
*C. camphora* (E.F.)	54.30 ± 1.09	1120
*C. verum* (Aq)	15.31 ± 0.47	N.A.
*C. verum* (Met)	17.92 ± 0.98	N.A.
Camphene	65.09 ± 0.08	0.147 (1.08 µM)
Cuminaldehyde	51.34 ± 0.56	0.214 (1.45 µM)

* Aq: aqueous extract, Met: methanol extract, H.F: hexane sub fraction, C.F: chloroform sub fraction, E.F: ethyl acetate sub fraction. ** Expressed as percentage decrease in the rate of enzyme activity with respect to control in absence of any inhibitor but in presence of equivalent amount of solvent. The concentration of samples (extract/pure compounds) used in the % Inhibition study was 0.1 mL of 1 mg/mL. Thiourea was taken as standard and % Inhibition was 71.47 ± 0.02.

**Table 2:** Summary of mode of urease inhibition by different samples.

Sample	Mode of Inhibition	K_i_ µg/mL
Aqueous (E)	Mixed	260
Methanol (E)	Non-Competitive	375
Hexane (F)	Competitive	175
Ethyl acetate (F) Camphene Cuminaldehyde	Mixed Competitive Mixed	370 195 (1.431 mM) 480 (3.238 mM)

E = extract, F = sub-fraction.

## Supplementary Materials

The following are available online, Figure S1: GC-MS chromatogram for Hexane extract of *C. camphora* leaves, Table S1: GC-MS Spectral analysis of Hexane fraction of *C. camphora* leaves.

## Sample Availability

Samples of the compounds are available from the authors.
