# Supplementary material for: Urease Inhibitory Kinetic Studies of Various Extracts and Pure Compounds from Cinnamomum Genus
Source: Molecules. 2021 Jun 22;26(13):3803. doi: 10.3390/molecules26133803 (PMC8270325; doi:10.3390/molecules26133803)
Supplement: Supplementary file 1 [file molecules-26-03803-s001.zip › molecules-1213070-supplementary.pdf]

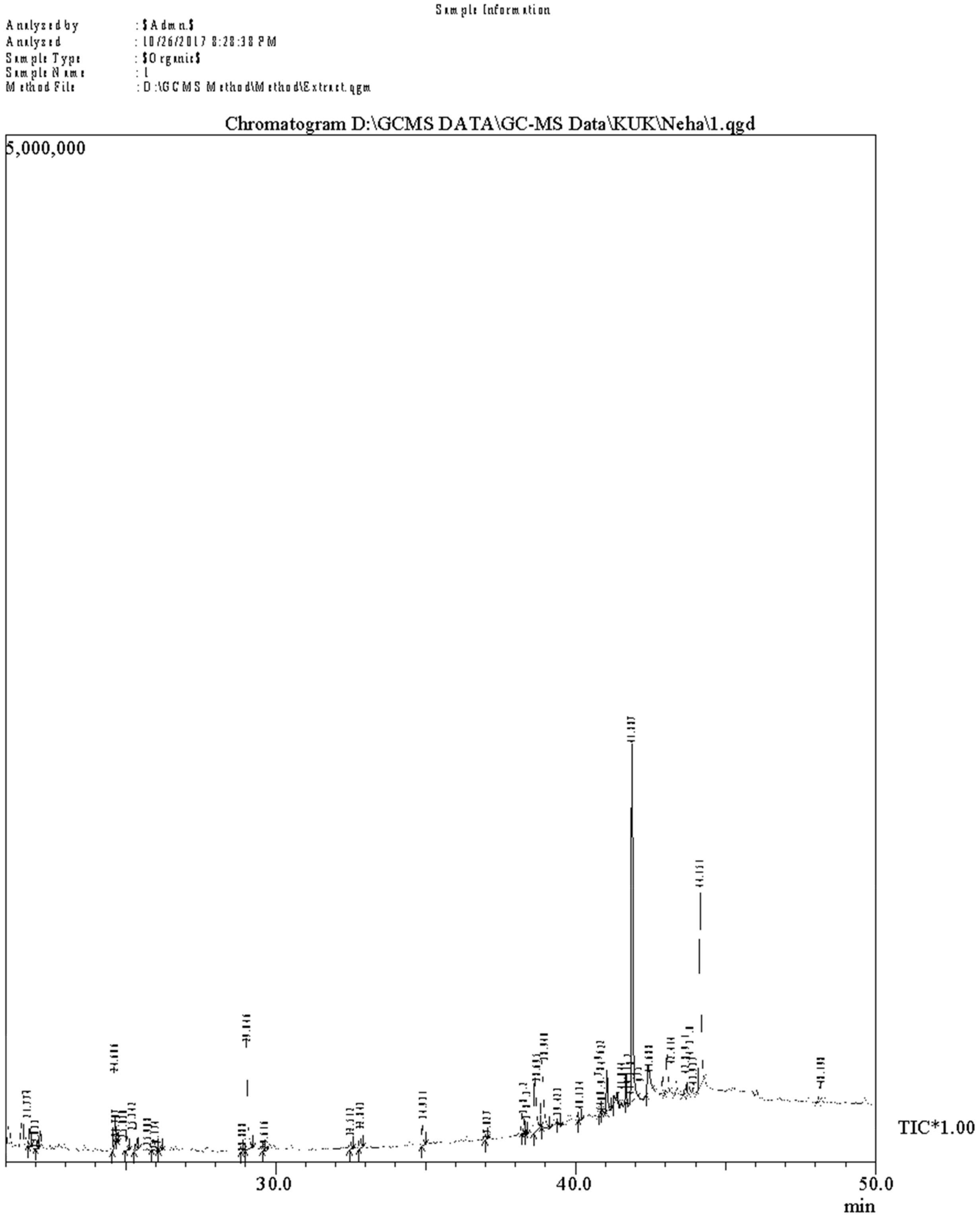


**Figure S1.** GC-MS chromatogram for Hexane extract of *C. camphora* leaves.

| Peak# | R.Time | Area | Area% | Name |
|---|---|---|---|---|
| 1 | 21.774 | 274361 | 1.05 | 10-12-Pentacosadiynoic acid |
| 2 | 22.033 | 79919 | 0.31 | (1R,7S,E)-7-Isopropyl-4,10-dimethylenecyclodec-5-enol |
| 3 | 24.606 | 1070284 | 4.12 | Neophytadiene |
| 4 | 24.707 | 89030 | 0.34 | 2-Pentadecanone, 6,10,14-trimethyl- |
| 5 | 25.030 | 305623 | 1.18 | Neophytadiene |
| 6 | 25.342 | 388684 | 1.49 | 3,7,11,15-Tetramethyl-2-hexadecen-1-ol |
| 7 | 25.908 | 220916 | 0.85 | 5,9,13-Pentadecatrien-2-one, 6,10,14-trimethyl- |
| 8 | 26.174 | 186620 | 0.72 | Hexacosanoic acid |
| 9 | 28.888 | 63462 | 0.24 | 6-Octadecenoic acid, methyl ester, (Z)- |
| 10 | 29.046 | 2175913 | 8.37 | Phytol |
| 11 | 29.616 | 100437 | 0.39 | 7-Octadecyne, 2-methyl- |
| 12 | 32.512 | 270615 | 1.04 | 4,8,12,16-Tetramethylheptadecan-4-olide |
| 13 | 32.843 | 343695 | 1.32 | 1,6,10,14,18,22-Tetracosahexaen-3-ol, 2,6,10,15,19,23- hexamethyl- |
| 14 | 34.931 | 385560 | 1.48 | 1,2-Benzenedicarboxylic acid |
| 15 | 37.027 | 83649 | 0.32 | Carbonic acid, eicosyl vinyl ester |
| 16 | 38.263 | 227975 | 0.88 | 1,6,10,14,18,22-Tetracosahexaen-3-ol,2,6,10,15,19,23-hexamethyl- |
| 17 | 38.357 | 167803 | 0.65 | Squalene |
| 18 | 38.695 | 1458862 | 5.61 | alpha-Tocospiro B |
| 19 | 38.940 | 1915635 | 7.37 | alpha-Tocospiro B |
| 20 | 39.423 | 92771 | 0.36 | Fumaric acid |
| 21 | 40.134 | 118510 | 0.46 | Octahydro-1-(2-Octyldecyl)-Pentalene |
| 22 | 40.792 | 80681 | 0.31 | Octahydro-1-(2-Octyldecyl)-Pentalene |
| 23 | 40.862 | 69276 | 0.27 | gamma-Tocopherol |
| 24 | 40.942 | 51956 | 0.20 | Oxirane, hexadecyl- |
| 25 | 41.044 | 741122 | 2.85 | gamma-Tocopherol |
| 26 | 41.275 | 132227 | 0.51 | Stigmast-5-en-3-ol |
| 27 | 41.389 | 72726 | 0.28 | 1-Bromooctdecane |
| 28 | 41.688 | 424021 | 1.63 | 9-Octadecenethioic acid, 12-hydroxy-, S-t-butyl ester |
| 29 | 41.887 | 6727366 | 25.87 | Vitamin E |
| 30 | 42.414 | 939445 | 3.61 | (+)-Sesamin |
| 31 | 42.891 | 304590 | 1.17 | Soleanesol |
| 32 | 43.039 | 778812 | 2.99 | (24R)-5-Ergosten-3beta-ol |
| 33 | 43.358 | 233181 | 0.90 | Pentacyclo[9.1.0.0(2,4).0(5,7).0(8,10)]dodecane, 3,3,6,6,9,9,12,12-octamethoxy- |
| 34 | 43.646 | 315179 | 1.21 | Nonacosan-14-one |
| 35 | 43.810 | 99714 | 0.38 | 1,6,10,14,18,22-Tetracosahexaen-3-ol, 2,6,10,15,19,23-hexmethyl-, (all-E)- |
| 36 | 43.937 | 126148 | 0.49 | Tetracontane-1,40-diol |
| 37 | 44.151 | 4481873 | 17.23 | gamma-Sitosterol |
| 38 | 48.198 | 409006 | 1.57 | Tris(2,4-di-tert-butylphenyl) phosphate |
| | | 26007647 | 100.00 | |

**Table S1.** GC-MS spectral analysis of hexane fraction of *C. camphora* leaves.
